# Prognostic value of chronic kidney disease in patients undergoing left atrial appendage occlusion

**DOI:** 10.1093/europace/euad315

**Published:** 2023-10-27

**Authors:** Domenico G Della Rocca, Michele Magnocavallo, Christoffel J Van Niekerk, Thomas Gilhofer, Grace Ha, Gabriele D'Ambrosio, Sanghamitra Mohanty, Carola Gianni, Jennifer Galvin, Giampaolo Vetta, Carlo Lavalle, Luigi Di Biase, Antonio Sorgente, Gian-Battista Chierchia, Carlo de Asmundis, Lukas Urbanek, Boris Schmidt, J Christoph Geller, Dhanunjaya R Lakkireddy, Moussa Mansour, Jacqueline Saw, Rodney P Horton, Douglas Gibson, Andrea Natale

**Affiliations:** Texas Cardiac Arrhythmia Institute, St.David's Medical Center, 3000 North I-35, Suite 720, Austin, TX 78705, USA; Heart Rhythm Management Centre, Postgraduate Program in Cardiac Electrophysiology and Pacing, Universitair Ziekenhuis Brussel-Vrije Universiteit Brussel, European Reference Networks Guard-Heart, Av. du Laerbeek 101, 1090 Jette, Brussels, Belgium; Texas Cardiac Arrhythmia Institute, St.David's Medical Center, 3000 North I-35, Suite 720, Austin, TX 78705, USA; Arrhythmology Unit, Ospedale Fatebenefratelli Isola Tiberina-Gemelli Isola, Rome, Italy; Interventional Electrophysiology, Scripps Clinic, 9898 Genesee Ave, La Jolla, CA 92037, USA; Division of Cardiology, Vancouver General Hospital, Vancouver, British Columbia, Canada; Cardiac Arrhythmia Service and Heart Center, Massachusetts General Hospital, Boston, MA, USA; Arrhythmia Section, Division of Cardiology, Zentralklinik Bad Berka, Bad Berka, Germany; Texas Cardiac Arrhythmia Institute, St.David's Medical Center, 3000 North I-35, Suite 720, Austin, TX 78705, USA; Texas Cardiac Arrhythmia Institute, St.David's Medical Center, 3000 North I-35, Suite 720, Austin, TX 78705, USA; Cardiac Arrhythmia Service and Heart Center, Massachusetts General Hospital, Boston, MA, USA; Heart Rhythm Management Centre, Postgraduate Program in Cardiac Electrophysiology and Pacing, Universitair Ziekenhuis Brussel-Vrije Universiteit Brussel, European Reference Networks Guard-Heart, Av. du Laerbeek 101, 1090 Jette, Brussels, Belgium; Department of Clinical, Internal, Anesthesiologist and Cardiovascular Sciences, Sapienza University of Rome, Viale del Policlinico 155, 00161 Rome, Italy; Texas Cardiac Arrhythmia Institute, St.David's Medical Center, 3000 North I-35, Suite 720, Austin, TX 78705, USA; Department of Medicine, Montefiore Medical Center, Albert Einstein College of Medicine, Bronx, NY, USA; Heart Rhythm Management Centre, Postgraduate Program in Cardiac Electrophysiology and Pacing, Universitair Ziekenhuis Brussel-Vrije Universiteit Brussel, European Reference Networks Guard-Heart, Av. du Laerbeek 101, 1090 Jette, Brussels, Belgium; Heart Rhythm Management Centre, Postgraduate Program in Cardiac Electrophysiology and Pacing, Universitair Ziekenhuis Brussel-Vrije Universiteit Brussel, European Reference Networks Guard-Heart, Av. du Laerbeek 101, 1090 Jette, Brussels, Belgium; Heart Rhythm Management Centre, Postgraduate Program in Cardiac Electrophysiology and Pacing, Universitair Ziekenhuis Brussel-Vrije Universiteit Brussel, European Reference Networks Guard-Heart, Av. du Laerbeek 101, 1090 Jette, Brussels, Belgium; Academy for Arrhythmias (FAFA), Abteilung für Kardiologie, Medizinische Klinik III, Agaplesion Markus Krankenhaus, Cardioangiologisches Centrum Bethanien, Frankfurt, Germany; Academy for Arrhythmias (FAFA), Abteilung für Kardiologie, Medizinische Klinik III, Agaplesion Markus Krankenhaus, Cardioangiologisches Centrum Bethanien, Frankfurt, Germany; Arrhythmia Section, Division of Cardiology, Zentralklinik Bad Berka, Bad Berka, Germany; Otto-von-Guericke University School of Medicine, Pziger Str. 44, 39120 Magdeburg, Germany; Kansas City Heart Rhythm Institute and Research Foundation, Overland Park, KS, USA; Cardiac Arrhythmia Service and Heart Center, Massachusetts General Hospital, Boston, MA, USA; Division of Cardiology, Vancouver General Hospital, Vancouver, British Columbia, Canada; Texas Cardiac Arrhythmia Institute, St.David's Medical Center, 3000 North I-35, Suite 720, Austin, TX 78705, USA; Interventional Electrophysiology, Scripps Clinic, 9898 Genesee Ave, La Jolla, CA 92037, USA; Texas Cardiac Arrhythmia Institute, St.David's Medical Center, 3000 North I-35, Suite 720, Austin, TX 78705, USA; Interventional Electrophysiology, Scripps Clinic, 9898 Genesee Ave, La Jolla, CA 92037, USA; Department of Cardiology, MetroHealth Medical Center, Case Western Reserve University School of Medicine, Cleveland, OH, USA

**Keywords:** Atrial fibrillation, Stroke, Watchman, Left atrial appendage, Chronic kidney disease

## Abstract

**Aims:**

Atrial fibrillation (AF) and chronic kidney disease (CKD) often coexist and share an increased risk of thrombo-embolism (TE). CKD concomitantly predisposes towards a pro-haemorrhagic state. Our aim was to evaluate the prognostic value of CKD in patients undergoing percutaneous left atrial appendage occlusion (LAAO).

**Methods and results:**

A total of 2124 consecutive AF patients undergoing LAAO were categorized into CKD stage 1+2 (*n* = 1089), CKD stage 3 (*n* = 796), CKD stage 4 (*n* = 170), and CKD stage 5 (*n* = 69) based on the estimated glomerular filtration rate at baseline. The primary endpoint included cardiovascular (CV) mortality, TE, and major bleeding. The expected annual TE and major bleeding risks were estimated based on the CHA_2_DS_2_-VASc and HAS-BLED scores. A non-significant higher incidence of major peri-procedural adverse events (1.7 vs. 2.3 vs. 4.1 vs. 4.3) was observed with worsening CKD (*P* = 0.14). The mean follow-up period was 13 ± 7 months (2226 patient–years). In comparison to CKD stage 1+2 as a reference, the incidence of the primary endpoint was significantly higher in CKD stage 3 (log-rank *P*-value = 0.04), CKD stage 4 (log-rank *P*-value = 0.01), and CKD stage 5 (log-rank *P*-value = 0.001). Left atrial appendage occlusion led to a TE risk reduction (RR) of 72, 66, 62, and 41% in each group. The relative RR of major bleeding was 58, 44, 51, and 52%, respectively.

**Conclusion:**

Patients with moderate-to-severe CKD had a higher incidence of the primary composite endpoint. The relative RR in the incidence of TE and major bleeding was consistent across CKD groups.

## Graphical Abstract

**Graphical Abstract:**
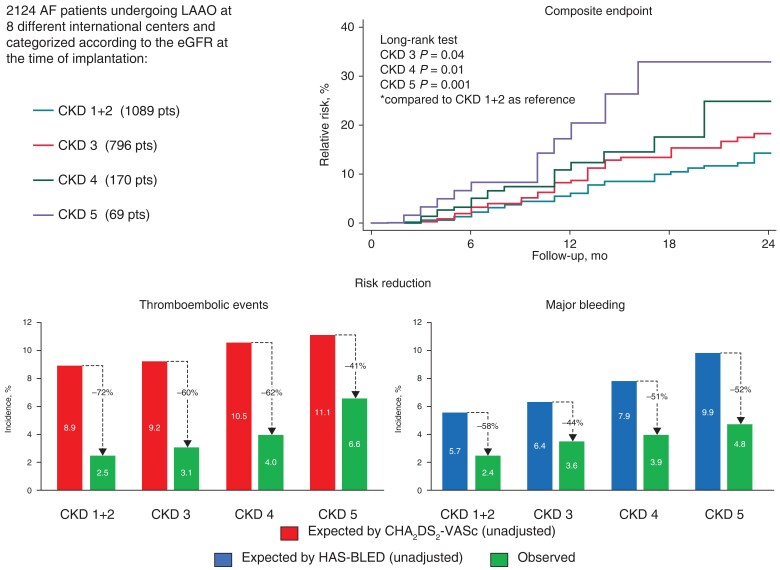

**What’s new?**

- We report the largest, real-world, multicentre cohort of LAAO patients categorized based on baseline kidney function.
- Left atrial appendage occlusion procedural success was high, irrespective of the severity of kidney dysfunction at the time of LAAO procedure.
- Left atrial appendage occlusion led to a relative risk reduction in the incidence of TE and major bleeding that was consistent across CKD groups, irrespective of their very different risk profiles at baseline.
- Left atrial appendage occlusion may provide an effective TE and bleeding prevention, irrespective of the baseline kidney function, when compared with the expected rates for patients with similar CHA_2_DS_2_-VASc and HAS-BLED scores.
- Future randomized studies comparing the efficacy of LAAO vs. oral anticoagulation in patients with CKD are warranted.

## Introduction

In high-risk patients with non-valvular atrial fibrillation (AF), stroke prevention via percutaneous left atrial appendage occlusion (LAAO) has been demonstrated to be non-inferior to oral anticoagulation with either vitamin K antagonists and direct oral anticoagulants (DOACs).^1^ The benefit of left atrial appendage (LAA) exclusion from systemic circulation has been further corroborated by the results of the recent Left Atrial Appendage Occlusion during Cardiac Surgery to Prevent Stroke (LAAOS III).^2^ This randomized trial showed a significant beneficial effect on thrombo-embolic (TE) risk in cardiac surgery candidates with AF undergoing concomitant appendage exclusion.

As a result of a growing body of evidence on the efficacy of LAAO and the remarkable safety profiles of currently available percutaneous devices,^3–5^ LAAO has now become a widespread therapy for stroke prophylaxis. Atrial fibrillation patients currently eligible for LAAO have multiple comorbid conditions, chronic kidney disease (CKD) being one of the most prevalent. Indeed, AF and CKD often coexist and are both associated with an increased risk of TE events and mortality, as they share common risk factors, including hypertension, coronary artery disease (CAD), and diabetes mellitus.^6–9^ Renal functional impairment concurrently contributes to an increase in haemorrhagic risk as kidney function declines. Chronic kidney disease-related haemorrhagic diathesis has a multifactorial pathophysiology (e.g. uraemia-related platelet dysfunction, altered von Willebrand factor, and endothelial functional impairment).

This bleeding tendency raises a therapeutic conundrum in patients with concomitant AF, as the potential benefit on stroke prophylaxis conferred by oral anticoagulation may be outweighed by an increased risk in major bleeding. In this perspective, LAAO may be a particularly attractive approach in this population, as it may confer TE protection without increasing the risk of bleeding.^10^ The objective of this registry was to elucidate the impact of CKD on procedural success and clinical outcomes after LAAO.

## Methods

### Patients

The registry included 2192 consecutive patients with non-valvular AF scheduled for percutaneous LAAO with a Watchman 2.5 device (Boston Scientific Corporation, Maple Grove, MN, USA) at eight different international centres (Austin, TX, USA; La Jolla, CA, USA; Kansas City, MO, USA; Vancouver, Canada; Boston, MA, USA; New York, NY, USA; Bad Berka, Germany; Frankfurt/Main, Germany) between December 2014 and June 2020. Eligibility criteria included ≥18 years of age with non-valvular AF who were unsuitable for long-term oral anticoagulation (previous major bleeding or contraindications for oral anticoagulation) and had a CHADS_2_ score of ≥2 or a CHA_2_DS_2_-VASc score of ≥3.

Clinical characteristics, procedural variables, and follow-up data were extracted from Institutional Review Board-approved, locally maintained, prospective databases. The Watchman implantation technique has been previously described elsewhere.^11,12^ Written informed consent was obtained from each patient before every interventional procedure.

### Study groups

Patients were categorized according to the estimated glomerular filtration rate (eGFR) at the time of implantation. The eGFR (mL/min/1.73 m^2^) was calculated using the Chronic Kidney Disease Epidemiology Collaboration (CKD-EPI) equation: 141 × min(serum creatinine/*κ*,1)*α* × max(serum creatinine/*κ*, 1) − 1.209 × 0.993Age × 1.018 [if female] × 1.159 [if black], where serum creatinine and Age are expressed in mg/dL and years, respectively, *κ* is 0.7 for females and 0.9 for males, and *α* is −0.329 for females and −0.411 for males.

Patients were divided into 4 study groups: CKD stage 1+2 (eGFR ≥ 60 mL/min/1.73 m^2^), CKD stage 3 (eGFR 30 to 59 mL/min/1.73 m^2^), CKD stage 4 (eGFR 15 to 29 mL/min/1.73 m^2^), and CKD stage 5 (eGFR < 15 mL/min/1.73 m^2^ or dialysis).

### Outcome measures

Implant success was defined as successful deployment of the device into the LAA.

The primary safety endpoint included any of the following major peri-procedural adverse events occurring within 7 days after the procedure: death, stroke, transient ischaemic attack (TIA), peripheral embolism, major peri-procedural bleeding, myocardial infarction, device embolization, acute heart failure, pericardial effusion requiring surgery or percutaneous drainage, retroperitoneal haematoma or other major vascular complications requiring surgical repair.

Overall peri-procedural adverse event rate was calculated including all the above-mentioned major peri-procedural complications plus groin haematoma and pericardial effusion not requiring intervention.

The primary efficacy endpoint included a composite of cardiovascular (CV) death, stroke, TIA, peripheral embolism, clinically significant bleeding during follow-up.

Clinically significant bleeding was defined according to the International Society of Thrombosis and Haemostasis criteria^13^ and included major and clinically relevant minor events.

The incidence of acute kidney injury (AKI) was assessed in all patients not receiving regular dialysis before the procedure and with creatinine assessment within 7 days before and between 24 and 48 h after the procedure.

Post-implant antithrombotic regiment and follow-up strategies were left to each operator’s preference.

### Definitions

Bleeding events were classified according to the International Society of Thrombosis and Haemostasis criteria as major, clinically relevant minor, and non-clinically relevant minor bleeding.^13^ Major bleeding included: fatal bleeding, symptomatic bleeding in a critical area or organ (e.g. intracranial), and/or bleeding causing a fall in haemoglobin ≥2 g/dL or requiring transfusion of ≥2 units of packed red blood cells or whole blood. Clinically relevant minor bleeding included bleeding requiring a clinical response, such as hospital admission, physician guided medical or surgical treatment, or need for antithrombotic therapy changes.

### Statistical analysis

For continuous variables, descriptive statistics were provided (number of available observations, mean, standard deviation), while median (interquartile range) was used for non-normal data. Categorical data were described as number (percentage). Comparisons among groups were performed using Pearson’s bivariate test and *χ*^2^ tests for categorical covariates; non-parametric test of Kruskal–Wallis was used to compare non-normally distributed continuous variable. For all tests, a *P*-value < 0.05 was considered statistically significant. Association between CKD stage and clinical endpoints was examined using the Cochran–Armitage trend test.

Odds ratios (ORs) and 95% confidence intervals (95% CIs) were calculated to estimate the association between CKD and other clinical findings (AKI, peri-procedural complications) using logistic regression analysis. Univariate and multivariate analyses were performed, and all the variables with a significant association (*P*-value < 0.10) in the univariate analysis were included in the multivariate analysis.

The Kaplan–Meier method was used to estimate cumulative event rates in the four groups. Differences in each group were compared using log-rank tests. CKD stage 1+2 was used as a reference to calculate the prognostic values of baseline renal function using a Cox regression hazard model. The expected annual TE and major bleeding risks were estimated based on the CHA_2_DS_2_-VASc and HAS-BLED scores and compared with the annualized observed risk aiming at calculating the % risk reduction (RR). Further details are reported in the Supplementary material.

## Results

### Study population

We enrolled 2192 consecutive patients with an indication to percutaneous LAA occlusion. Device implant was unsuccessful in 68 (3.1%) patients; baseline characteristics and predictors of implant success are reported in Supplementary material online, *Tables S1 and S2*.

The final study cohort with successful Watchman implantation included 2124 patients (mean age: 77 ± 8 years; 62.7% males; CHA_2_DS_2_-VASc: 4.7 ± 1.4; HAS-BLED: 3.5 ± 1.0). Among them, 51.3% (*n* = 1089) were categorized as CKD stage 1+2, 37.5% (*n* = 796) as CKD stage 3, 8.0% (*n* = 170) as CKD stage 4, and 3.2% (*n* = 69) as CKD stage 5. Of those with end-stage CKD, 72.5% (*n* = 50) were on haemodialysis.

Baseline and procedural data are depicted in *Tables 1* and *2*. Differences in gender, vascular disease, chronic heart failure (CHF), and other risk factors for stroke and bleeding were statistically significant. These variations in baseline characteristics led to significant differences in CHA_2_DS_2_-VASc (*P* < 0.001) and HAS-BLED (*P* < 0.001) among groups.

**Table 1:** Baseline characteristics

Demographics	CKD stage 1+2 (*n* = 1089)	CKD stage 3 (*n* = 796)	CKD stage 4 (*n* = 170)	CKD stage 5 (*n* = 69)	*P*-value
Age, years	75.5 (70.2–80.4)	79 (73.5–84)	80.1 (75.8–85)	76.8 (69.1–81.4)	**<0**.**001**
Female	629 (57.8)	123 (15.5)	27 (15.9)	14 (20.3)	**<0**.**001**
Black ethnicity	42 (4.3)	34 (4.3)	12 (7.1)	9 (13.0)	**0**.**001**
BMI, kg/m^2^	27.5 (23.9–31.2)	27.4 (24.2–31.2)	26.4 (23.3–31.1)	28.1 (23.3–31.7)	**<0**.**001**
*CHA_2_DS_2_-VASc score*					
Median (Q1**–**Q3)	4 (3–6)	5 (4–6)	5 (4–6)	5 (4–6)	**<0**.**001**
Score					
** **3	273 (25.1)	192 (24.1)	25 (14.7)	7 (10.2)	**0**.**001**
** **4	309 (28.3)	186 (23.4)	26 (15.3)	17 (24.6)	**0**.**001**
** ≥**5	508 (46.6)	418 (52.5)	119 (70.0)	45 (65.2)	**<0**.**001**
*HAS-BLED score*					
Median (Q1**–**Q3)	3 (3–4)	3 (3–4)	4 (3–5)	4 (4–5)	**<0**.**001**
Score					
** **2	236 (21.7)	113 (14.2)	23 (13.5)	6 (8.7)	**<0**.**001**
** **3	422 (38.7)	299 (37.6)	37 (21.8)	9 (13.0)	**<0**.**001**
** **4	308 (28.3)	266 (33.4)	56 (32.9)	17 (24.6)	0.06
** ≥**5	123 (11.3)	118 (14.8)	54 (31.8)	37 (53.7)	**<0**.**001**
*Risk factors for stroke and bleeding*					
CHF	265 (24.3)	289 (36.3)	91 (53.5)	40 (58.0)	**<0**.**001**
Hypertension	975 (89.5)	731 (91.8)	157 (92.4)	68 (98.6)	**0**.**03**
Age **≥**75	571 (52.4)	548 (68.9)	134 (78.8)	41 (59.4)	**<0**.**001**
Age 65–74	417 (50.7)	223 (28.0)	30 (17.6)	17 (24.6)	**<0**.**001**
Diabetes mellitus	309 (28.4)	257 (32.3)	70 (41.2)	40 (58)	**<0**.**001**
Hx. of stroke/TIA	428 (39.3)	377 (47.4)	81 (47.6)	28 (40.6)	**0**.**003**
** **Stroke	262 (24.1)	235 (29.5)	52 (30.6)	20 (29.0)	**0**.**04**
** **TIA	166 (15.2)	142 (17.9)	29 (17.1)	8 (11.6)	0.32
Vascular disease	454 (41.7)	373 (46.9)	79 (46.5)	40 (58.0)	**0**.**01**
Abnormal liver function	50 (4.6)	35 (4.4)	13 (7.6)	15 (21.7)	**<0**.**001**
Hx. of major bleeding	574 (52.7)	491 (61.7)	108 (63.5)	40 (58.0)	**0**.**001**
** **Intracranial bleeding	125 (11.5)	113 (14.2)	17 (10.0)	5 (7.3)	0.12
** **GI bleeding	310 (28.5)	260 (32.7)	65 (38.2)	28 (40.6)	**0**.**01**
** **Other	139 (12.7)	118 (14.8)	26 (15.3)	7 (10.1)	0.43
Hx. of minor bleeding	149 (13.7)	111 (13.9)	24 (14.1)	15 (21.7)	0.32
** **GI bleeding	48 (4.4)	38 (4.8)	9 (5.3)	9 (13.0)	**0**.**02**
** **Epistaxis	61 (5.6)	44 (5.5)	13 (7.6)	5 (7.3)	0.68
** **Other	40 (3.7)	29 (3.6)	2 (1.2)	1 (1.4)	0.29
Labile INR	160 (14.7)	70 (8.8)	30 (17.6)	14 (20.3)	**<0**.**001**
Drug interactions	393 (36.1)	340 (42.7)	66 (38.8)	24 (34.8)	**0**.**03**
Alcohol	90 (8.3)	90 (11.3)	13 (7.6)	6 (8.7)	0.13
CAD	189 (17.4)	186 (23.4)	26 (15.3)	20 (29.0)	**<0**.**001**
LVEF, % (range)	55 (50–60)	55 (42–60)	45 (35–55)	47 (37.5–55)	**<0**.**001**

Baseline characteristics and risk factors of the study population categorized based on the CKD stage at baseline. Values are *n* (%) or median (interquartile range).

BMI, body mass index; CAD, coronary artery disease; CHF, congestive heart failure; CKD, chronic kidney disease; GI, gastrointestinal; INR, International Normalized Ratio; LVEF, left ventricular ejection fraction; Q1 and Q3, first and third quartile (25th and 75th percentiles); TIA, transient ischaemic attack.

**Table 2:** Peri-procedural adverse events

Procedural data	CKD stage 1+2 (*n* = 1089)	CKD stage 3 (*n* = 796)	CKD stage 4 (*n* = 170)	CKD stage 5 (*n* = 69)	*P*-value
Watchman size					
** **21** **mm	147 (13.5)	92 (11.6)	19 (11.2)	6 (8.7)	0.42
** **24** **mm	319 (29.3)	197 (24.7)	44 (25.8)	21 (30.4)	0.15
** **27** **mm	314 (28.8)	252 (31.7)	52 (30.6)	22 (31.9)	0.60
** **30** **mm	173 (15.9)	154 (19.3)	36 (21.2)	14 (20.3)	0.13
** **33** **mm	136 (12.5)	101 (12.7)	19 (11.2)	6 (8.7)	0.76
Procedural duration, min	78 (51–104)	84 (55–112.5)	91 (72–120)	101 (68.5–139.5)	**<0**.**001**
Contrast, ml	80 (50–120)	90 (50–130)	50 (30–90)	50 (27.5–130)	**<0**.**001**
Hospital length of stay, d	1 (1–1)	1 (1–1)	1 (1–1)	1 (1–2)	0.10
*Device- and procedural-related events*					
Death	0	0	1 (0.6)	0	–
Stroke	3 (0.3)	0	0	0	–
TIA	1 (0.1)	0	0	0	–
Air embolism	0	0	0	0	–
MI	1 (0.1)	1 (0.1)	0	0	–
Major bleeding	2 (0.2)	3 (0.4)	2 (1.2)	1 (1.4)	0.11
Device embolization	0	1 (0.1)	0	0	–
Acute heart failure	0	2 (0.3)	1 (0.6)	1 (1.4)	–
Pericardial effusion	16 (1.5)	12 (1.5)	3 (1.8)	1 (1.4)	0.99
** **Requiring surgery	2 (0.2)	1 (0.1)	0	0	–
** **Requiring percutaneous drainage	7 (0.6)	6 (0.8)	2 (1.2)	1 (1.4)	0.79
** **No intervention	7 (0.6)	5 (0.6)	1 (1.2)	0	0.88
Vascular complication	9 (0.8)	14 (1.8)	5 (2.9)	2 (2.9)	0.06
** **Retroperitoneal haematoma	3 (0.3)	4 (0.5)	1 (0.6)	0	0.74
** **Groin haematoma	6 (0.6)	10 (1.3)	4 (2.4)	2 (2.9)	**0**.**04**
*Composite major adverse events*	19 (1.7)	18 (2.3)	7 (4.1)	3 (4.3)	0.14
*Overall adverse events*	32 (2.9)	33 (4.1)	12 (7.1)	6 (8.7)	**0**.**01**

Procedure-related complications occurring within 7 days after the procedure. Values are *n* (%) or median (interquartile range).

MI, myocardial infarction; TIA, transient ischaemic attack.

### Peri-procedural complications

Procedural duration (*Table 2*) significantly increased in parallel with CKD severity among groups (83 ± 42 min vs. 88 ± 44 min vs. 99 ± 43 min vs. 102 ± 46 min, *P* < 0.001).

No differences were documented regarding hospital length of stay.

A non-significant higher incidence of major peri-procedural adverse events (1.7 vs. 2.3 vs. 4.1 vs. 4.3) was observed with worsening baseline kidney function (*P* = 0.14; *Table 2*).

Cox regression analysis for the association between major peri-procedural complications and CKD stages is reported in *Table 3*.

**Table 3:** Predictors of major peri-procedural complications

Predictors of MAE	Univariate analysis			Multivariate analysis		
	OR	95% CI	*P*-value	OR	95% CI	*P*-value
Age **≥** 75 years	1.229	(0.719–2.099)	0.45	–	–	–
BMI (per 1** **kg/m^2^ increase)	0.992	(0.950–1.035)	0.70	–	–	–
Female gender	1.171	(0.698–1.966)	0.55	–	–	–
CHF	1.476	(0.879–2.480)	0.14	–	–	–
Hypertension	3.010	(0.730–12.415)	0.13	–	–	–
Diabetes	1.216	(0.751–1.969)	0.32	–	–	–
History of stroke/TIA/SE	1.305	(1.010–1.685)	**0**.**04**	1.049	(0.763–1.443)	0.77
Vascular disease	1.058	(0.635–1.764)	0.83			
History of CAD	2.181	(1.272–3.741)	**0**.**01**	2.053	(1.183–3.563)	**0**.**01**
Medication usage predisposing to bleeding	2.016	(1.210–3.358)	**0**.**01**	1.416	(0.822–2.441)	0.21
CHA**2**DS_2_-VASc score **≥** 6	1.941	(1.161–3.245)	**0**.**01**	1.313	(0.697–2.475)	0.40
HAS-BLED score **≥** 5	3.696	(2.180–6.265)	**<0.001**	2.937	(1.613–5.348)	**<0.001**

Baseline renal function	OR	95% CI	*P*-value
CKD stage 1+2	1	–	–
CKD stage 3	1.237	(0.615–1.975)	0.78
CKD stage 4	1.921	(0.863–4.275)	0.23
CKD stage 5	2.334	(0.795–6.854)	0.12

Univariate and multivariate analysis for predictors of major procedural adverse events (primary safety endpoint).

BMI, body mass index; CAD, coronary artery disease; CHF congestive heart failure; CI, confidence interval; MAE, major adverse events; OR, odds ratio; SE, systemic embolism; TIA, transient ischaemic attack.

At multivariate analysis, history of CAD (OR: 2.053; 95% CI: 1.183–3.563; *P* = 0.01) and HAS-BLED ≥5 (OR: 2.937; 95% CI: 1.613–5.348; *P* < 0.001) were the only independent predictors of major complications (*Table 3*).

Among minor complications, the incidence of groin haematoma in each group was 0.6, 1.3, 2.4, and 2.9, respectively (*P* = 0.04).

Overall peri-procedural complications (major and minor) were significantly different among groups (2.9 vs. 4.1 vs. 7.1 vs. 8.7; *P* = 0.01).

Data on the incidence and predictors of AKI are reported in the supplemental results and in Supplementary material online, *Tables S3* and *S4*.

### Primary efficacy endpoint

**Figure 1:**
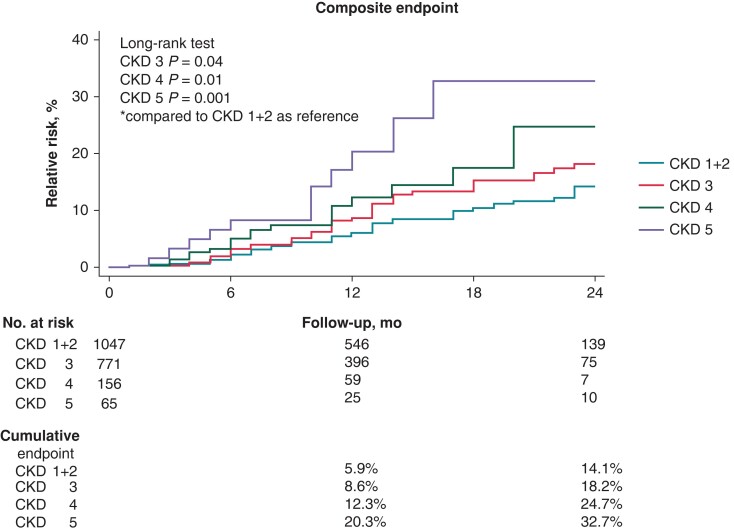
Primary composite endpoint. Cumulative incidence function for the primary endpoint of CV mortality, stroke, TIA, peripheral TE events, major bleeding. CKD, chronic kidney disease; CV, cardiovascular; TE, thrombo-embolic; TIA, transient ischaemic attack.

Follow-up data were available in 2039 patients. The mean follow-up period was 13 ± 7 months (2226 patient–years, PY) and was not different among groups (*P* = 0.41). There were 170 (8.3%) patients who met the primary composite endpoint of CV death, stroke, TIA, peripheral embolism, clinically significant bleeding for a total of 177 clinical events (8.0 events/100PY) (see Supplementary material online, *Table S5*). The incidence of the primary endpoint at 1 year and 2 years significantly increased with worsening CKD across the four study groups (1-year cumulative incidence: 5.9 vs. 8.6 vs. 12.3 vs. 20.3%; 2-year cumulative incidence: 14.1 vs. 18.2 vs. 24.7 vs. 32.7; *Figure 1*). In comparison to CKD stage 1+2 as a reference, the incidence of the primary endpoint over the entire follow-up period was significantly higher in CKD stage 3 (log-rank *P*-value = 0.04), CKD stage 4 (log-rank *P*-value = 0.01), and CKD stage 5 (log-rank *P*-value = 0.001; Cochran–Armitage trend test *z* = −2.902; *P*-value = 0.004).

In the Cox regression analysis (*Table 4*), CKD stage 3 (hazard ratio, HR: 1.404; 95% CI: 1.009–1.953; *P* = 0.04), CKD stage 4 (HR: 1.384; 95% CI: 1.068–1.793; *P* = 0.01), CKD stage 5 (HR: 1.436; 95% CI: 1.162–1.775; *P* = 0.001), as well as AKI (2.367; 95% CI: 1.272–4.404; *P* = 0.01) were associated with an increased risk of the primary endpoint in the multivariate model. The influence of different post-procedure antithrombotic strategies on the composite outcome is reported in Supplementary material online, *Table S6*.^12^

**Table 4:** Cox regression analysis for the association between composite endpoint and clinical findings

Predictors of composite endpoint	Univariate analysis			Multivariate analysis		
	HR	95% CI	*P*-value	HR	95% CI	*P*-value
Age **≥** 75 years	1.340	(0.972–1.848)	0.07	–	–	–
Female gender	1.173	(0.865–1.590)	0.31	–	–	–
CHF	1.534	(1.129–2.084)	**0**.**01**	1.365	(0.873–2.134)	0.17
Hypertension	1.586	(0.810–3.103)	0.18	–	–	–
Diabetes	1.467	(1.076–1.999)	**0**.**02**	1.475	(0.950–2.291)	0.08
History of TE	1.164	(1.002–1.353)	**0**.**05**	1.089	(0.854–1.389)	0.49
Vascular disease	1.593	(1.177–2.157)	**0**.**004**	1.483	(0.963–2.286)	0.07
History of CAD	1.186	(0.814–1.728)	0.38	–	–	–
CHA_2_DS_2_-VASc Score **≥** 4	1.411	(1.040–1.913)	**0**.**03**	0.904	(0.528–1.548)	0.71
HAS-BLED score **≥** 5	1.537	(1.068–2.213)	**0**.**02**	1.023	(0.581–1.801)	0.94
Liver disfunction	1.386	(0.752–2.555)	0.30	–	–	–
AKI	2.209	(1.148–4.252)	**0**.**001**	2.367	(1.272–4.404)	**0**.**01**

Baseline renal function	HR	95% CI	*P*-value
CKD stage 1+2	1	–	–
CKD stage 3	1.404	(1.009–1.953)	**0**.**04**
CKD stage 4	1.384	(1.068–1.793)	**0**.**01**
CKD stage 5	1.436	(1.162–1.775)	**0**.**001**

AKI, acute kidney injury; CAD, chronic artery disease; CI, confidence of interval; CKD, chronic kidney disease; CHF, congestive heart failure; HR, hazard ratio; TE, thrombo-embolic.

### Thrombo-embolic events, clinically relevant bleeding, CV mortality

**Figure 2:**
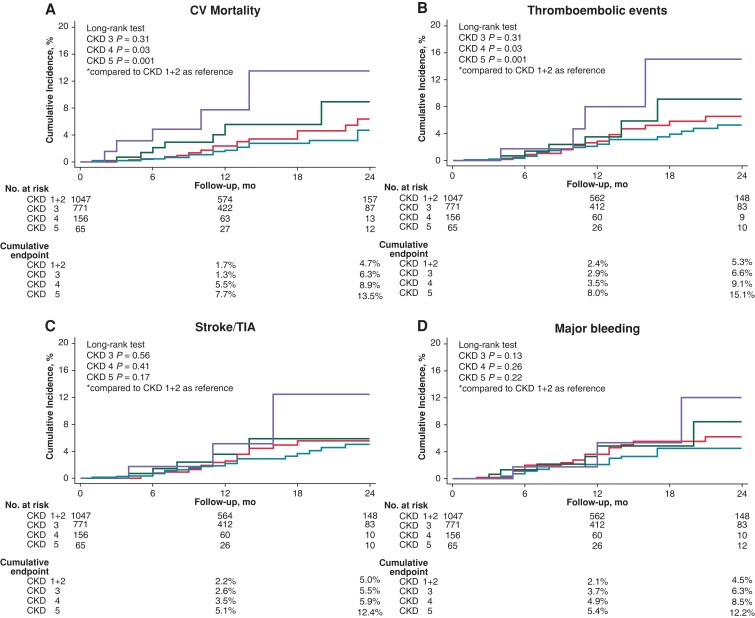
Secondary endpoints. Kaplan–Meier survival curves for *A*) CV mortality, *B*) TE events, *C*) stroke/TIA, and *D*) major bleeding. CKD, chronic kidney disease; CV, cardiovascular; TIA, transient ischaemic attack.

A linear increase in event rates for TE events, stroke/TIA, and clinically relevant bleeding was observed among the four groups; however, the differences were not significant (*Figure 2*).

The annualized rates of TE events were 2.5% in CKD stage 1+2, 3.1% in CKD stage 3, 4.0% in CKD stage 4, and 6.6% in CKD stage 5, respectively (see Supplementary material online, *Table S5*). The comparison between the expected annual TE and major bleeding risks and the annualized observed risk are reported in the supplemental results and in Supplementary material online, *Figure S2*.

The annualized CV mortality rates (see Supplementary material online, *Table S5*) increased in parallel with CKD severity among groups (1.9 vs. 2.5 vs. 4.5 vs. 8.0; *P* = 0.016; Cochrane–Armitage test: *z* = −3.903; *P*-value = <0.001). A significant difference in CV mortality was observed among CKD stage 4 and CKD stage 5 (log-rank *P*-value = 0.03 and 0.001, respectively), compared to CKD stage 1+2 patients (*Figure 2*).

Minor bleeding occurred in 2.8% of patients with CKD stage 1+2, 4.4% with CKD stage 3, 5.8% with CKD stage 4, 9.2% with CKD stage 5 (*P*-value = 0.01; Cochrane–Armitage test: *z* = −3.022; *P* = 0.003; Supplementary material online, *Table S5*).

Data on all-cause mortality are reported in the supplemental results and in Supplementary material online, *Figure S1*.

## Discussion

This study provides a comprehensive analysis on the safety and long-term efficacy of LAAO based on a real-world, multicentre experience in a large cohort of AF patients categorized based on their kidney function at the time of the appendage occlusion procedure.

The main findings are the following:

- Left atrial appendage occlusion patients with eGFR < 60 mL/min/1.73 m^2^ represented 48.7% (*n* = 1035) of our cohort; among them, 239 (23.1%) patients had severely decreased renal function or organ failure.
- The burden of cardiovascular comorbid conditions was significantly higher in patients at more advanced stages of CKD; nonetheless, procedural success was high, irrespective of the severity of kidney dysfunction at the time of LAAO procedure.
- A higher incidence of major peri-procedural adverse events was observed with worsening baseline kidney function; however, the differences were not statistically significant.
- Chronic kidney disease classification was a strong predictor of the primary composite endpoint of CV mortality, stroke, TIA, peripheral TE events, and major bleeding.
- Left atrial appendage occlusion provided an effective TE and bleeding prevention, irrespective of the baseline kidney function, when compared with the expected rates for patients with similar CHA_2_DS_2_-VASc and HAS-BLED scores.

### Procedural success and safety

In our cohort, Watchman implantation procedure was highly successful (96.9%); this finding is concordant with other recent reports on the same occlusion device.^14–19^

Additionally, Watchman implantation was a fairly safe procedure, as demonstrated by a mean major complication rate of 2.2%. Chronic kidney disease severity predicted neither successful device deployment nor major peri-procedural complications. Notably, despite a different cardiovascular and metabolic risk profile at baseline, the differences in major complications were not statistically significant (*P* = 0.14). This observation is consistent with a previous study in 1014 patients undergoing LAAO with an Amplatzer Cardiac Plug.^20,21^ Although major complications were similar among CKD groups, the rate of overall complications was significantly higher in patients with more advanced stages of CKD. A similar significant increment associated with CKD severity was also observed for minor vascular complications/groin haematoma, which was likely the main determinant for the higher prevalence of total peri-procedural complications. Two large studies have recently reported higher procedural complications and longer hospital stays in CKD patients included in the National Inpatient Sample and Nationwide Readmissions Databases.^22,23^ The partial discrepancy among our findings and these previous observations may be attributed to the high level of experience with LAAO of the operators involved in our study. Additionally, these studies were limited to first generation devices; next generation LAA occluders (e.g. Watchman FLX) have recently demonstrated a remarkable safety profile^3–5,24,25^ and may be particularly beneficial in frail populations, such as those with severe/end-stage CKD.

### TE events, bleeding, mortality

Chronic kidney disease affects approximately 13–15% of the population worldwide and is a well-known, independent risk factor for cardiovascular disease and all-cause mortality.^26–28^ Chronic kidney disease shares several risk factors with AF and its prevalence is markedly increased in AF patients and vice versa. Although CKD is a well-known independent predictor of TE events in AF patients, too little is still known about the optimal strategy for stroke prophylaxis in patients with severely impaired kidney function.^29^ Chronic kidney disease patients also display a graded, increased bleeding risk as renal function declines (e.g. the relative risk of intracranial haemorrhage in chronic dialysis patients is >10 times higher than that of the general population). The resulting concomitant predisposition to both thrombosis and bleeding in AF patients with CKD emphasizes the need for a better understanding of the risk/benefit ratio of currently available pharmacological and non-pharmacological strategies for stroke prophylaxis.^7^

Percutaneous LAAO has been proven to be non-inferior to oral anticoagulation for preventing AF-related major TE and bleeding events; furthermore, recent long-term data showed a significant reduction of non-procedural bleeding with LAAO.^10^

However, there have been only a few studies assessing the impact of kidney function on peri-procedural and clinical outcomes in patients undergoing LAAO; their main limitations are the small number of patients with more advanced forms of kidney dysfunction and the relatively short follow-up duration.^7,20,22,23,30^ Two large studies using the Nationwide Readmissions^22^ and National Inpatient Sample^23^ Databases have recently investigated the association between kidney function and in-hospital/short-term outcomes; however, no data were provided on the incidence of TE events and bleeding in the long-term. Other studies have previously attempted to assess the association between kidney function and LAAO efficacy but their observations were limited by the small number of patients with more advanced stages of CKD.^20,30^

The present analysis included 1035 patients with at least moderate loss of kidney function (eGFR < 60 mL/min/1.73 m^2^); Among them, 239 (23.1%) patients had severe CKD or failure (eGFR < 30 mL/min/1.73 m^2^), 50 (20.9%) of whom in chronic dialysis. On average, patients were followed for 13 ± 7 months. The degree of kidney dysfunction was associated with mid/long-term prognosis of patients undergoing LAAO. Specifically, the incidence of the primary endpoint over the entire follow-up period was significantly higher in CKD stages 3, 4, and 5, compared to CKD stage 1+2. Baseline kidney function was also associated with a significantly higher incidence of CV mortality in advanced-stage CKD (stages 4 and 5).

In our report, we have also highlighted trends towards a higher incidence of stroke/TIA and major bleeding, which increase in parallel with CKD severity; however, these differences were not statistically significant.

Of note, the TE RR was 72% in CKD stage 1+2 patients vs. 41% among patients with end-stage CKD. In interpreting these outcome findings, it is of utmost importance to contextualize our observations within the very different risk profiles of the study groups at baseline. Specifically, patients in dialysis have a significantly higher burden of cardiovascular comorbidities (e.g. hypertension, dyslipidaemia, heart failure) independently predisposing to cerebrovascular events, irrespective of the presence of AF. In patients on OAC, either DOACs or vitamin K antagonists, a paradoxical increased rate of ischaemic stroke has been described among patients with a moderate/severe decrease in eGFR, as well as end-stage renal disease.^31,32^ Unlike OACs, our analysis showed that LAAO provides protection against TE events and major bleeding at any stage of CKD, as compared to the expected rates estimated based on the CHA_2_DS_2_-VASc and HAS-BLED of the population. Specifically, LAAO efficacy was consistent across groups at different severity of renal functional impairment, irrespective of a different burden of cardiovascular comorbid conditions at baseline.

### Limitations

(1) Although data were extracted from a database of prospectively collected data, the *post-hoc* nature of this analysis introduces all the inherent limitations related to its design. (2) Our observations on the prevalence of peri-procedural AKI were based on a single creatinine assessment at 24–48 h after the procedure, which might have led to an underestimation of the incidence of AKI. (3) All patients included in the study received a Watchman device; therefore, our findings cannot be generalized to other occlusion devices. (4) The calculation of eGFR using the CKD-EPI equation may be limited by age; however, this method proved superior to other equations in terms of both kidney function estimation and risk prediction. (5) The number of patients with advanced CKD was small, compared to the other groups. Nonetheless, this study includes the largest cohort of patients with severe and end-stage CKD. (6) Our cohort included only patients with LAAO, and no comparison was performed with other pharmacological strategies for stroke prophylaxis.

## Conclusions

In this real-world, multicentre cohort of LAAO patients categorized based on baseline kidney function, CKD was associated with a higher incidence of overall, but not major, peri-procedural complications. Patients with moderate-to-severe CKD also had a higher incidence of the primary endpoint of CV mortality, stroke, TIA, peripheral TE, and major bleeding. The relative RR in the incidence of TE and major bleeding was consistent across CKD groups. Future randomized studies comparing the efficacy of LAAO vs. oral anticoagulation in patients with CKD are warranted.

## Supplementary Material

euad315_Supplementary_Data

## Data Availability

The data underlying this article will be shared on reasonable request to the corresponding author.
