# Supplementary material for: Prognostic value of chronic kidney disease in patients undergoing left atrial appendage occlusion
Source: Europace. 2023 Oct 27;25(11):euad315. doi: 10.1093/europace/euad315 (PMC10653166; doi:10.1093/europace/euad315)

**SUPPLEMENTAL MATERIALS**

**Prognostic Value of Chronic Kidney Disease in Patients Undergoing**

**Left Atrial Appendage Occlusion**

**Supplemental Methods**

Definitions

Statistical Analysis

**Supplemental Results**

Incidence and Predictors of Acute Kidney Injury

All-Cause Mortality

**Additional Tables**

Supplemental Table 1

Supplemental Table 2

Supplemental Table 3

Supplemental Table 4

Supplemental Table 5

Supplemental Table 6

**Additional Figure**

Supplemental Figure 1

Supplemental Figure 2

**Supplemental Methods**

**Definitions.**

AKI was defined according to the Acute Kidney Injury Network criteria as an increase in serum creatinine of ≥0.3 mg/dL or of ≥50% over the course of 48 hours post-procedure. Patients meeting this definition were further categorized in three stages: stage 1, increase in serum creatinine ≥ 0.3 mg/dl (≥ 26.4 μmol/l) or of 1.5 to 2 times compared to baseline; stage 2, increase in serum creatinine of 2 to 3 times compared to baseline; stage 3, increase in serum creatinine of > 3 times compared to baseline or serum creatinine ≥ 4.0 mg/dl [≥ 354 μmol/l] with an acute increase of at least 0.5 mg/dl [44 μmol/l].

**Statistical Analysis.**

Normal distribution of all continuous variables (age, BMI, CHA_2_DS_2_-VASc Score, HAS-BLED Score, LVEF, Contrast Volume Injection and Procedural Time) was checked by visual methods (Q-Q plot and histogram) and by significance test (Kolmogorov-Smirnov normality test and Shapiro-Wilk’s test). The proportional-hazards assumptions were verified with the use of Schoenfeld residuals. A univariate Cox regression analysis was performed to obtain the hazard ratio (HR) for the cumulative endpoint. Thereafter, a multivariate analysis was performed using the variables with p-values <0.10 in the univariate analysis to examine their independent associations. The correlation between the composite endpoint and the post-procedure antithrombotic strategy was evaluated by means of time-to-event analysis with Cox frailty models, using warfarin and aspirin as the reference strategy.

**Supplemental Results**

*Incidence and Predictors of Acute Kidney Injury*

Post-procedural AKI was documented in 68 (5.5%) of 1234 patients with creatinine determination between 24 and 48 hours post-LAAO. Baseline characteristics of patients categorized according to the occurrence of AKI are reported in the supplemental table 3.

AKI was associated with higher CHA_2_DS_2_-VASc (5.3 ± 1.4 vs. 4.8 ± 1.4; p=0.001) and HAS-BLED (3.8 ± 0.9 vs. 3.5 ± 1.0; p=0.01) scores at baseline, as well as a higher prevalence of diabetes mellitus (p=0.04) and CHF (p<0.001).

At multivariate analysis (supplemental table 4), the only independent predictors of AKI were male gender (OR: 2.044; 95% CI: 1.198 – 3.487; p=0.01), CKD stage 4/5 (OR: 1.998; 95% CI: 1.018 – 3.923; p=0.04), and CHF (OR: 2.286; 95% CI: 1.328 – 3.934; p=0.01).

*TE Events and Clinically Relevant Bleeding*

The annualized rates of TE events were 2.5% in CKD stage 1+2, 3.1% in CKD stage 3, 4.0% in CKD stage 4, and 6.6% in CKD stage 5, respectively (supplemental table 5). Compared to the unadjusted, estimated annual rates of TE events for patients with similar CHA_2_DS_2_-VASc scores, LAAO led to a risk reduction of 72%, 66%, 62%, and 41% in each group, respectively (supplemental figure 2A). The relative risk reduction in the incidence of major bleeding was 58%, 44%, 51%, and 52%, respectively (supplemental figure 2B).

*All-Cause Mortality*

Overall, 154 (7.6%) patients died of any cause during the study period (7.3 deaths/100PY). The annualized rate of all-cause mortality was 5.7% (n= 63) in patients with CKD stage 1+2, 8.4% (n= 66) with CKD stage 3, 10.5% (n= 16) with CKD stage 4, and 14.5% (n= 9) with CKD stage 5 (p= 0.02). Kaplan-Meier curves and 1- and 2-year cumulative incidence of all-cause mortality are reported in Supplemental Figure 1.

**Additional Tables**

**Supplemental Table 1.** **Patients with/without Procedural Success.** Comparison of Baseline Characteristics among Patients with/without Procedural Success. Values are n (%) or median [interquartile range]. *BMI: Body Mass Index; CAD: Coronary Artery Disease; CHF: Congestive Heart Failure; GI: Gastrointestinal; LVEF: Left Ventricular Ejection Fraction; Q1 and Q3: first and third quartile (25th and 75th percentiles); TIA: Transient Ischemic Attack.*

| Demographics | Success  (n=2124) | Failure  (n=68) | p-value |
| --- | --- | --- | --- |
| Age, yrs | 77.1 [71.6 – 82.6] | 77.1 [71.9 – 83.9] | 0.29 |
| Male | 1331 (62.7) | 52 (76.51) | **0.02** |
| Black ethnicity | 97 (4.6) | 2 (2.9) | 0.52 |
| BMI, kg/m^2^ | 27.4 [23.9 – 31.2] | 26.3 [21.9 – 30.9] | 0.26 |
| *CHA_2_DS_2_-VASc Score* | | | |
| Median [Q1-Q3] | 5 [4-6] | 5 [4-6] | 0.11 |
| Score |  |  |  |
| 3 | 497 (23.4) | 11 (16.2) | 0.16 |
| 4 | 538 (25.3) | 18 (26.5) | 0.82 |
| ≥5 | 1089 (51.3) | 39 (57.3) | 0.32 |
| *HAS-BLED Score* | | | |
| Median [Q1-Q3] | 3 [3-4] | 3.5 [3-4] | 0.24 |
| Score |  |  |  |
| 2 | 378 (17.8) | 11 (16.2) | 0.73 |
| 3 | 767 (36.1) | 23 (33.8) | 0.70 |
| 4 | 647 (30.5) | 19 (27.9) | 0.65 |
| ≥5 | 332 (15.6) | 15 (22.1) | 0.15 |
| *Risk Factors for Stroke and Bleeding* | | | |
| CHF | 685 (32.3) | 38 (55.9) | **< 0.001** |
| Hypertension | 1931 (90.9) | 66 (97.1) | 0.08 |
| Age ≥75 | 1294 (60.9) | 43 (63.2) | 0.70 |
| Age 65-74 | 686 (32.3) | 22 (32.4) | 1 |
| Diabetes Mellitus | 676 (31.8) | 22 (32.4) | 0.92 |
| Hx. Of Stroke/TIA | 914 (43.0) | 32 (47.1) | 0.51 |
| Stroke | 569 (26.8) | 20 (29.4) | 0.63 |
| TIA | 345 (16.2) | 12 (17.7) | 0.75 |
| Vascular Disease | 946 (44.5) | 32 (47.1) | 0.68 |
| Abnormal Liver Function | 113 (5.3) | 5 (7.4) | 0.46 |
| Hx. of Major Bleeding | 1213 (57.1) | 41 (60.3) | 0.60 |
| Intracranial Bleeding | 260 (12.2) | 3 (4.4) | **0.05** |
| GI Bleeding | 663 (31.2) | 24 (35.3) | 0.48 |
| Other | 290 (13.7) | 14 (20.6) | 0.10 |
| Hx. Of Minor Bleeding | 299 (14.1) | 14 (20.6) | 0.59 |
| GI Bleeding | 104 (4.9) | 5 (7.4) | 0.36 |
| Epistaxis | 123 (5.8) | 5 (7.4) | 0.59 |
| Other | 72 (3.4) | 4 (5.8) | 0.27 |
| Drug Interactions | 823 (38.7) | 27 (39.7) | 0.86 |
| Alcohol | 199 (9.4) | 6 (8.8) | 0.89 |
| CAD | 421 (19.8) | 9 (8.8) | 0.8 |
| LVEF, % | 55 [45-65] | 45 [39-57.5] | **0.02** |

| Predictors of Failure | Univariate analysis | | | Multivariate analysis | | |
| --- | --- | --- | --- | --- | --- | --- |
|  | **OR** | **95% CI** | **p-value** | **OR** | **95% CI** | **p-value** |
| Age ≥ 75 years | 1.037 | [0.631 – 1.705] | 0.89 |  |  |  |
| BMI (per 1 kg/m^2^ increase) | 1.364 | [0.935 – 1.017] | 0.24 |  |  |  |
| Female Gender | 0.506 | [0.287 – 0.893] | **0.02** | 0.455 | [0.223– 0.926] | **0.03** |
| CKD 4-5 | 1.914 | [1.030 – 3.558] | **0.04** | 1.054 | [0.490– 2.268] | 0.89 |
| CHF | 2.758 | [1.694 – 4.492] | **< 0.001** | 1.863 | [1.034 – 3.356] | **0.04** |
| Hypertension | 3.380 | [0.821 – 13.907] | 0.09 | 3.625 | [0.482 – 27.250] | 0.21 |
| Diabetes | 1.025 | [0.612 – 1.718] | 0.93 |  |  |  |
| History of Stroke/TIA/TE | 1.095 | [0.859 – 1.395] | 0.47 |  |  |  |
| Vascular Disease | 1.111 | [0.684 – 1.802] | 0.67 |  |  |  |
| Prior Major Bleeding | 1.145 | [0.699 – 1.876] | 0.59 |  |  |  |
| CHA_2_DS_2_VASc Score ≥ 6 | 1.502 | [0.913 – 2.472] | 0.10 | 1.161 | [0.622 – 2.167] | 0.64 |
| HAS-BLED Score ≥ 5 | 1.551 | [0.863 – 2.785] | 0.14 |  |  |  |

**Supplemental Table 2.** **Predictor of Procedural Failure.** Univariate and Multivariate Analysis for Predictors of Procedural Failure. *BMI: Body Mass Index; CHF: Congestive Heart Failure; CI: Confidence Interval; eGFR: estimated Glomerular Filtration Rate; OR: Odds Ratio; TE: Thromboembolism; TIA: Transient Ischemic Attack.*

**Supplemental Table 3.** **Patients with/without AKI.** Baseline characteristics of patients with AKI. Values are n (%) or median [interquartile range]. *AKI: Acute Kidney Injury;* *BMI: Body Mass Index; CAD: Coronary Artery Disease; CHF: Congestive Heart Failure; eGFR: estimated glomerular filtration rate; GI: Gastrointestinal; IQR: interquartile range; LVEF: Left Ventricular Ejection Fraction; Q1 and Q3: first and third quartile (25th and 75th percentiles); TIA: Transient Ischemic Attack.*

| Demographics | Non-AKI  (n=1166) | AKI  (n=68) | p-value |
| --- | --- | --- | --- |
| Age, yrs | 77.7 [72.5 – 83.4] | 81.0 [73.1 – 84.9] | 0.14 |
| Male | 715 (57.9) | 51 (75.0) | **0.03** |
| Black ethnicity | 68 (5.8) | 4 (5.9) | 0.98 |
| BMI, kg/m^2^ | 27.4 [23.7-31.0] | 26.9 [24.3-32.0] | 0.74 |
| *CHA_2_DS_2_-VASc Score* | | | |
| Median [Q1-Q3] | 5 [4-6] | 5.5 [4-6] | **0.001** |
| Score |  |  |  |
| 3 | 237 (20.3) | 9 (13.2) | 0.16 |
| 4 | 318 (27.3) | 9 (13.2) | **0.01** |
| ≥5 | 611 (52.4) | 50 (73.6) | **< 0.001** |
| *HAS-BLED Score* | | | |
| Median [Q1-Q3] | 3 [3-4] | 3 [3-4] | **0.01** |
| Score |  |  |  |
| 2 | 169 (14.5) | 5 (7.4) | 0.10 |
| 3 | 453 (38.8) | 15 (22.1) | **0.01** |
| 4 | 365 (31.3) | 37 (54.4) | **< 0.001** |
| ≥5 | 179 (15.4) | 11 (16.1) | 086 |
| *AKI* | | | |
| Type I | - | 63 (92.6) |  |
| Type II | - | 4 (5.9) |  |
| Type III | - | 1 (1.5) |  |
| *Risk Factors for Stroke and Bleeding* | | | |
| CHF | 372 (31.9) | 40 (58.8) | **< 0.001** |
| Hypertension | 1091 (93.6) | 66 (97.1) | 0.25 |
| Age ≥75 | 755 (64.8) | 44 (64.7) | 1 |
| Age 65-74 | 353 (30.3) | 20 (29.4) | 0.89 |
| Diabetes Mellitus | 345 (29.6) | 28 (41.2) | **0.04** |
| Hx. Of Stroke/TIA | 473 (40.6) | 34 (50.0) | 0.12 |
| Stroke | 292 (25.1) | 19 (27.9) | 0.59 |
| TIA | 181 (15.5) | 15 (22.1) | 0.15 |
| Vascular Disease | 545 (46.7) | 30 (44.1) | 0.67 |
| Abnormal Liver Function | 54 (4.6) | 4 (5.9) | 0.64 |
| Hx. of Major Bleeding | 651 (55.8) | 39 (57.4) | 0.81 |
| Intracranial Bleeding | 135 (11.6) | 6 (8.8) | 0.49 |
| GI Bleeding | 344 (29.5) | 22 (32.4) | 0.62 |
| Other | 172 (14.7) | 11 (16.2) | 0.75 |
| Hx. Of Minor Bleeding | 148 (12.7) | 13 (19.1) | 0.13 |
| Drug Interactions | 467 (40.1) | 35 (51.5) | 0.06 |
| Alcohol | 109 (9.3) | 8 (11.8) | 0.51 |
| CAD | 115 (9.9) | 11 (16.2) | 0.09 |
| LVEF, % | 53 [40-62.3] | 50 ± 14 [25-70] | 0.58 |
| *Procedural Data* | | | |
| Contrast Volume, mL | 80 [50 – 130] | 87.5 [40 – 130] | 0.17 |
| Contrast Volume/eGFR [IQR] | 1.4 [0.8-2.3] | 1.4 [0.9-2.7] | 0.20 |

**Supplemental Table 4.** **Predictors and Stages of AKI.** Univariate and multivariate analysis for predictors of AKI (**panel A**) and incidence of AKI among groups (**panel B**). *AKI: Acute Kidney Injury, CAD: Coronary Artery Disease, CHF: Congestive Heart Failure, CI: Confidence Interval, eGFR: estimated Glomerular Filtration Rate, INR: International Normalized Ratio, NSAID: Nonsteroidal Anti-Inflammatory Drug, OR: Odds Ratio.*

| A. Variables | Univariate analysis | | | Multivariate analysis | | |
| --- | --- | --- | --- | --- | --- | --- |
|  | **OR** | **95% CI** | **p-value** | **OR** | **95% CI** | **p-value** |
| Age ≥ 75 years | 0.998 | [0.598 – 1.665] | 0.99 |  |  |  |
| Male Gender | 1.829 | [1.120 – 2.986] | **0.02** | 2.044 | [1.198 – 3.487] | **0.01** |
| CKD 4-5 | 2.102 | [1.136 – 3.892] | **0.02** | 1.998 | [1.018 – 3.923] | **0.04** |
| CHF | 3.049 | [1.852 – 5.019] | **< 0.001** | 2.286 | [1.328 – 3.934] | **0.01** |
| Hypertension | 2.269 | [0.545 – 9.442] | 0.26 |  |  |  |
| Diabetes | 1.666 | [1.011 – 2.744] | **0.04** | 1.319 | [0.756 – 2.268] | 0.34 |
| History of CAD | 1.764 | [0.899 – 3.459] | 0.10 |  |  |  |
| CHA_2_DS_2_VASc Score ≥ 6 | 2.370 | [1.449 – 3.875] | **0.001** | 1.461 | [0.835 – 2.556] | 0.18 |
| Administration of NSAID | 1.588 | [0.973 – 2.591] | 0.06 | 1.519 | [0.903 – 2.556] | 0.12 |
| HAS-BLED Score ≥ 5 | 1.064 | [0.547 – 2.069] | 0.86 |  |  |  |
| Contrast Volume ≥ 200 ml | 1.782 | [0.907 – 3.504] | 0.09 | 1.853 | [0.923 – 3.721] | 0.08 |
|  |  |  |  |  |  |  |

| B. Stage | CKD 1 + 2  (n=625) | | CKD 3  (n=467) | | CKD 4  (n=125) | | CKD 5  (n=17) | | P-value |
| --- | --- | --- | --- | --- | --- | --- | --- | --- | --- |
|  | **Events** | **%** | **Events** | **%** | **Events** | **%** | **Events** | **%** |  |
| AKI | 25 | 4 | 29 | 6.2 | 12 | 9.6 | 2 | 11.8 | **0.04** |
| *Type I* | 22 | 3.5 | 28 | 6 | 11 | 8.8 | 2 | 11.8 | **0.03** |
| *Type II* | 3 | 0.5 | 1 | 0.2 | - | - | - | - | - |
| *Type III* | - | - | - | - | 1 | 0.8 | - | - | - |

**Supplemental Table 5:** **Clinical Outcomes.** Number of Events and Annualized Event Rates for the Primary and Secondary Endpoints. Values are n (%) or median [interquartile range]. *AKI: Acute Kidney Injury; CKD: Chronic Kidney Disease; CV: Cardiovascular; GI: Gastrointestinal; IC: Intracranial; SE: Systemic Embolism; TE: Thromboembolic; TIA: Transient Ischemic Attack.*

**Supplemental Table 6.** **Post-Procedure Antithrombotic Strategies and Associated Risk of Primary Outcome.** VKA plus Aspirin was considered as the reference group. *CI: Confidence Interval, DAPT: Dual Antiplatelet Therapy; DOAC: Direct Oral Anticoagulant; HR: Hazard Ratio; VKA: Vitamin K Antagonist.*

| Post-Implant Antithrombotic Regimen | CKD 1 + 2  (n=1047) | | | | CKD 3  (n=771) | | | | CKD 4  (n=156) | | | | CKD 5  (n=65) | | | |
| --- | --- | --- | --- | --- | --- | --- | --- | --- | --- | --- | --- | --- | --- | --- | --- | --- |
|  | **n** | **HR** | **CI** | **p-value** | **n** | **HR** | **CI** | **p-value** | **n** | **HR** | **CI** | **p-value** | **n** | **HR** | **CI** | **p-value** |
| VKA + Aspirin | 298 | 1 |  |  | 192 | 1 |  |  | 28 | 1 |  |  | 23 | 1 |  |  |
| DOAC + Aspirin | 387 | 0.67 | [0.41-0.96] | 0.04 | 177 | 0.51 | [0.20-1.31] | 0.16 | 41 | 0.71 | [0.14-3.53] | 0.68 |  |  |  |  |
| VKA | 108 | 0.73 | [0.31-1.35] | 0.25 | 152 | 0.96 | [0.40-2.27] | 0.92 | 33 | 0.89 | [0.11-2.87] | 0.81 | 21 | 1.07 | [0.21-5.31] | 0.94 |
| DOAC | 149 | 0.79 | [0.34-1.64] | 0.38 | 106 | 0.66 | [0.27-1.57] | 0.35 | 32 | 0.56 | [0.06-5.37] | 0.61 |  |  |  |  |
| DAPT | 105 | 1.12 | [0.49-2.58] | 0.79 | 144 | 1.11 | [0.47-2.61] | 0.82 | 22 | 1.46 | [0.33-6.53] | 0.62 | 21 | 1.20 | [0.23-6.12] | 0.83 |

**Supplemental Figure 1. Overall Mortality.** Cumulative incidence function for overall mortality. *CKD: chronic kidney disease.*

**Supplemental Figure 2. Expected and Annualized Rates of TE Events and Major Bleeding.** Annualized TE event rates vs expected (unadjusted) rates estimated based on the CHA_2_DS_2_-VASc (**panel A**) and annualized major bleeding vs expected (unadjusted) rates estimated based on the HAS-BLED (**panel B**). *CKD: chronic kidney disease.*


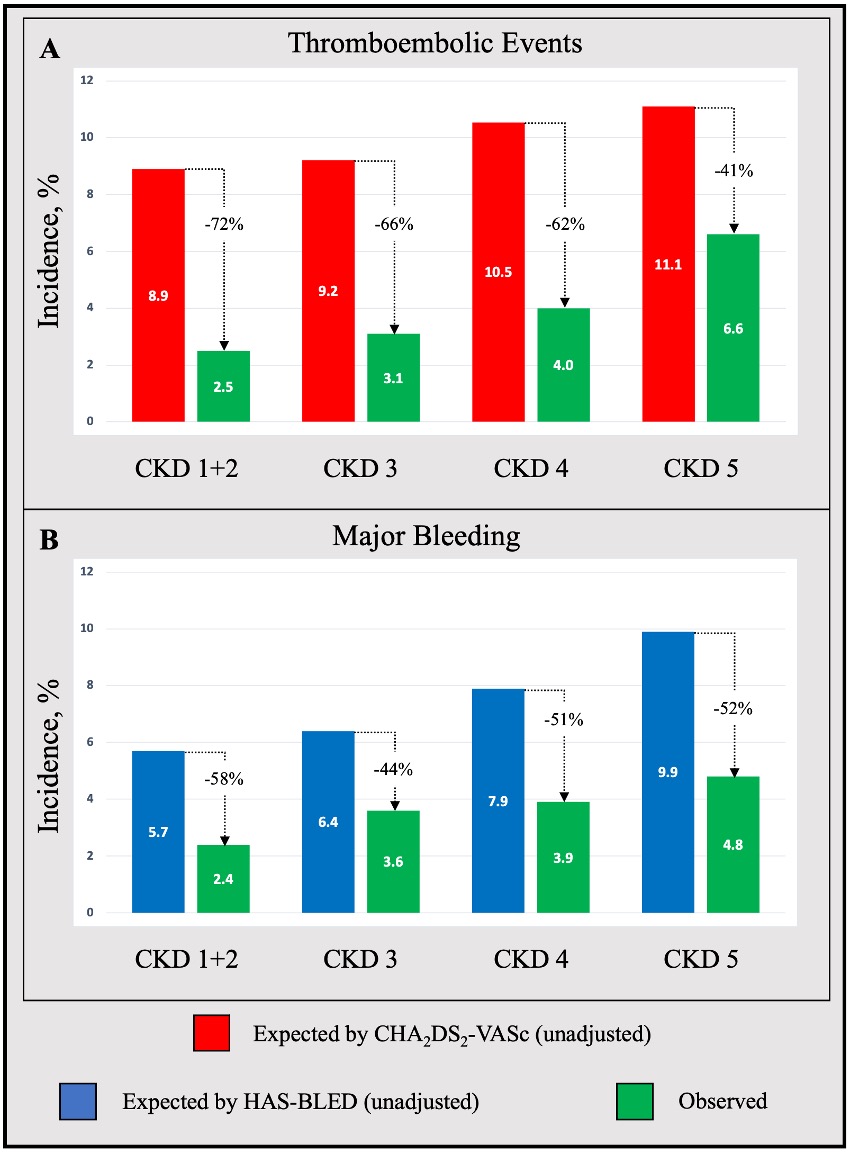

Supplement: euad315_Supplementary_Data [file euad315_supplementary_data.zip › Supplemental Materials.docx]
