# Supplementary figures and images for: Prognostic value of chronic kidney disease in patients undergoing left atrial appendage occlusion
Source: Europace. 2023 Oct 27;25(11):euad315. doi: 10.1093/europace/euad315 (PMC10653166; doi:10.1093/europace/euad315)

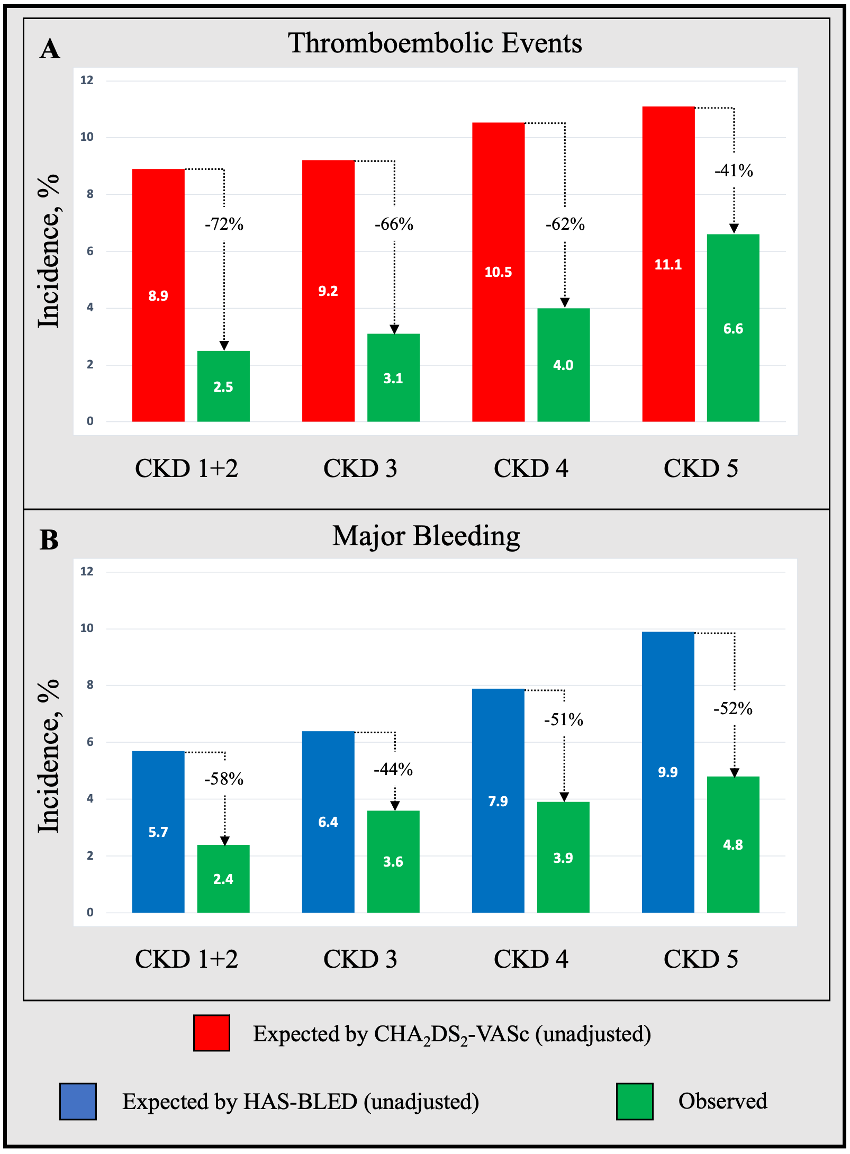

Supplement: euad315_Supplementary_Data [file euad315_supplementary_data.zip › Supplemental Figure 2.png]

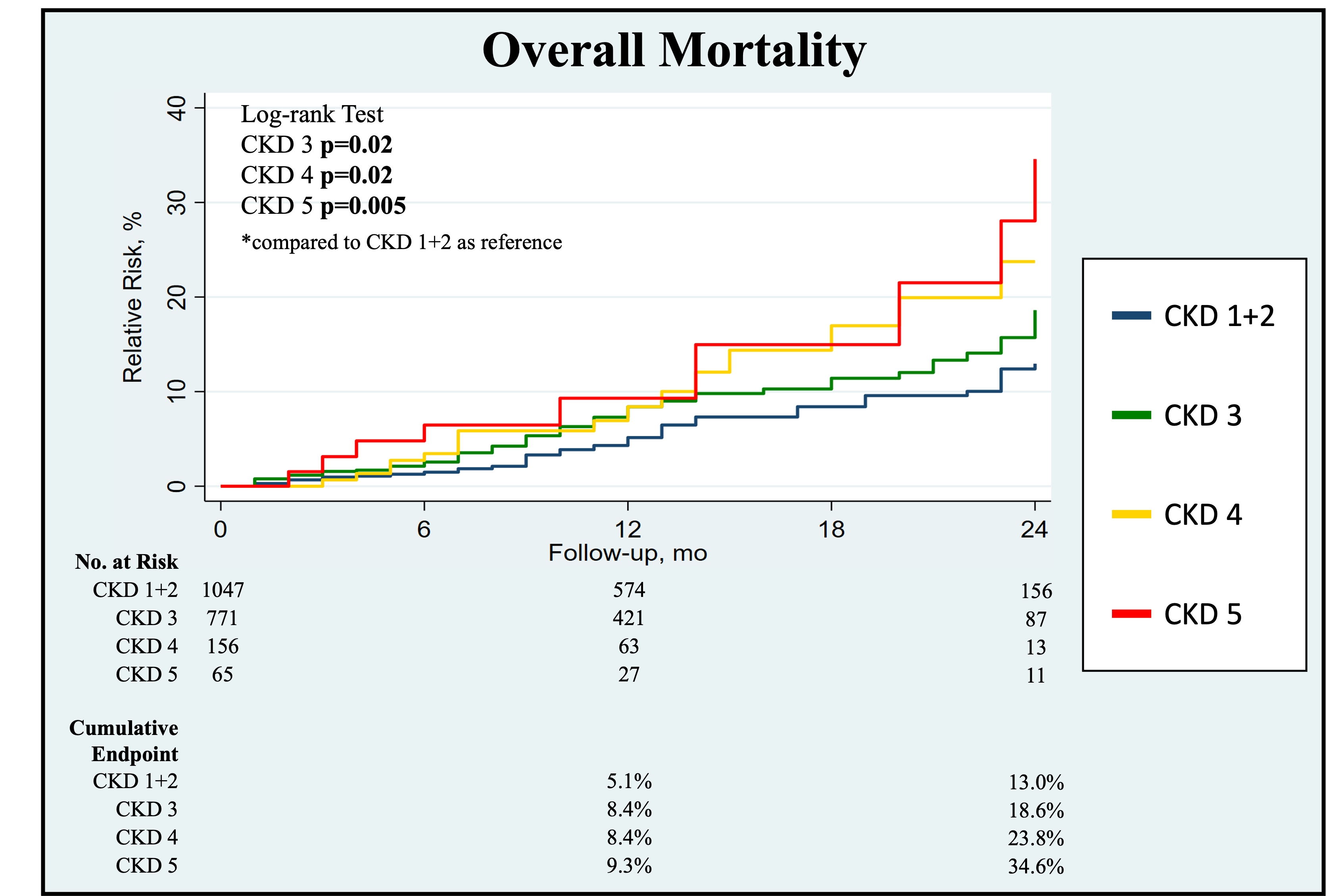

Supplement: euad315_Supplementary_Data [file euad315_supplementary_data.zip › Supplemental Figure 1.png]
